# Outcomes of patients with peripheral T-cell lymphoma in first complete remission: data from three tertiary Asian cancer centers

**DOI:** 10.1038/s41408-017-0030-y

**Published:** 2017-12-15

**Authors:** Tiffany Tang, Lay Poh Khoo, Cindy Lim, Jun Soo Ham, Seok Jin Kim, Huangming Hong, Colin Phipps, Yuh Shan Lee, Miriam Tao, Richard Quek, Mohamad Farid, Tongyu Lin, Won Seog Kim, Soon Thye Lim

**Affiliations:** 1 Division of Medical Oncology, National Cancer Centre Singapore, Singapore, Singapore; 2 Division of Clinical Trials and Epidemiological Sciences, National Cancer Centre Singapore, Singapore, Singapore; 3 Division of Hematology-Oncology, Department of Medicine, Samsung Medical Center, Sunkyunkwan University School of Medicine, Seoul, Korea; 4 Department of Medical Oncology, Sun Yat-Sen University Cancer Center, Guangzhou, People’s Republic of China; 5 State Key Laboratory of Oncology in Southern China, Collaborative Innovation Center of Cancer Medicine, Sun Yat-Sen University Cancer Centre Guangzhou, Guangzhou, People’s Republic of China; 6 Department of Hematology, Singapore General Hospital, Singapore, Singapore; 7 Oncology Academic Clinical Program, Duke-NUS Graduate Medical School, Singapore, Singapore; 8 Department of Health Sciences and Technology, SAIHST, Sunkyunkwan University, Seoul, Korea

Peripheral T-cell lymphomas (PTCL) represent a heterogenous group of aggressive non-Hodgkin’s lymphoma, with poorer treatment outcomes compared to that of their B-cell counterparts, using conventional chemotherapy^1^. Despite the lack of randomized data, upfront high-dose chemotherapy (HDC) and autologous stem cell transplantation (ASCT) has been associated with better treatment outcomes^2, 3^ and various guidelines now recommend that patients with chemosensitive PTCL should undergo upfront HDC/ASCT^4^. Consistently, both retrospective and prospective studies suggest that such a strategy appears to benefit patients with responding disease, especially those in first complete remission (CR1)^5^. However, it is conceivable that PTCL patients who do achieve CR1 may have more favorable survival outcomes, regardless of the treatment they received. Thus, we performed a retrospective analysis of PTCL patients who attained CR1 following first-line induction therapy to determine the factors that would impact their survival outcomes, including the role of upfront HDC/ASCT.

Prospectively maintained T-cell lymphoma databases from the National Cancer Centre Singapore (NCCS)/Singapore General Hospital (SGH), Samsung Medical Centre, South Korea, and Sun Yat Sen University Cancer Centre, China, were retrospectively reviewed after approval from the institutional review boards of the individual institutions. We included patients with the following histological subtypes: PTCL-not otherwise specified (PTCL-NOS), angioimmunoblastic T-cell lymphoma (AITL), anaplastic lymphoma kinase (ALK)-negative anaplastic large cell lymphoma (ALCL), and monomorphic epitheliotropic intestinal T-cell lymphoma (MEITL)^6, 7^, previously known as type II enteropathic T-cell lymphoma, treated with curative intent. To standardize the effect of treatment on outcome, we excluded patients with ALK-positive ALCL because their first-line treatment would not include considerations for upfront HDC/ASCT^4^ and patients with natural-killer/T-cell lymphoma whose treatment may have included upfront allogeneic stem cell transplantation^8^. Upfront HDC/ASCT was not standard practice in any of the participating institutions and left to the discretion of the primary physician or tumor board decisions. We also excluded patients with composite lymphomas, cutaneous T-cell lymphomas and patients who were not treated with curative intent. We then reviewed the clinical characteristics, treatment and survival outcomes of patients who achieved CR1. Patients who had partial response, stable disease and progression of disease were excluded from the analysis. Treating physicians determined the end of treatment response assessments. The exact modality was as per institutional standards, which was either computed tomography or positron-emission tomography scans.

Progression-free survival (PFS) was defined as the interval from diagnosis to progression, relapse or death. Overall survival (OS) was defined as the interval from diagnosis to death from any cause. Survival estimates were calculated using the Kaplan−Meier method. Survival curves were compared using the log-rank test. Univariate and multivariate analyses were performed using the Cox proportional hazards model to assess the association between several prognostic factors such as age, stage, international prognostic index (IPI)^9^, prognostic index for T-cell lymphoma (PIT)^10^ and treatment, including the receipt of upfront HDC/ASCT, with survival outcomes. For multivariate analysis, variable selection was performed using the forward selection method, with selection criteria of *P* < 0.05 for inclusion into the final multivariate model. All variables used in the univariate analysis were entered into the forward selection procedure, except for IPI and PIT scores as the individual components of these scores were already included. Two-sided *p*-values less than 0.05 were considered statistically significant. All analyses were performed in Stata (version 14.2, StataCorp, Texas, USA).

There were 114, 96, and 92 patients respectively in the Singapore, South Korea, and China PTCL database. A total of 175 patients were included in our analysis based on the inclusion and exclusion criteria; 57 from Singapore, 62 from South Korea, and 56 from China, resulting in an overall CR1 rate of 57.9%. Patients in the Singapore cohort were diagnosed between October 1998 and December 2015, patients in the Korean cohort were diagnosed between August 2007 and March 2014, and patients in the Chinese cohort were diagnosed between September 2007 and June 2016.

In this study, the median age was 53 years old, 63% were male and the majority of patients had an ECOG performance status one or less. The most common subtype of PTCL was PTCL-NOS accounting for 42% of the cohort, followed by AITL 33%, ALK negative ALCL 22%, and MEITL 3%. The majority of patients received anthracycline-based chemotherapy and 18% underwent upfront HDC/ASCT. Non-anthracycline-based chemotherapy included ICE (ifosfamide, carboplatin, etoposide) (3%), ESHAP (etoposide, steroids, cytarabine, cisplatin) (1%), Gemcitabine-based regimens (2%), and other regimens (2%) including alemtuzumab-DHAP (steroids, cytarabine, cisplatin) and high-dose methotrexate with procarbazine, and vincristine. In this cohort of patients, the ALK-negative ALCL patients were younger (median age 35 years), they were more likely to have stage I/II disease, normal lactate dehydrogenase (LDH) and less likely to have bone marrow involvement compared to the other subtypes of PTCL in this study. None of the ALK-negative ALCL patients underwent upfront HDC/ASCT (Table 1).

**Table 1:** Patient characteristics overall and by histological subtype

Characteristic	Overall (*n* = 175)	MEITL (*n* = 6)	AITL (*n* = 57)	PTCL-NOS (*n* = 74)	ALK- ALCL (*n* = 38)
Age, years					
Median	53	61.5	57	53	35
Range	16–85	43–66	28–85	16–78	16–75
Age, years (grouped)					
≤ 60	119 (68)	3 (50)	34 (60)	50 (68)	32 (84)
> 60	56 (32)	3 (50)	23 (40)	24 (32)	6 (16)
Sex					
Female	65 (37)	2 (33)	25 (44)	26 (35)	12 (32)
Male	110 (63)	4 (67)	32 (56)	48 (65)	26 (68)
B symptoms					
Present	76 (43)	3 (50)	31 (54)	29 (39)	13 (34)
Absent	92 (53)	2 (33)	25 (44)	43 (58)	22 (58)
Unknown	7 (4)	1 (17)	1 (2)	2 (3)	3 (8)
Stage					
I	28 (16)	2 (33)	3 (5)	16 (22)	7 (18)
II	22 (13)	1 (17)	1 (2)	10 (14)	10 (26)
III	57 (33)	0 (0)	26 (46)	19 (26)	12 (32)
IV	61 (35)	2 (33)	27 (47)	27 (36)	5 (13)
Unknown	7 (4)	1 (17)	0 (0)	2 (3)	4 (11)
I / II	50 (29)	3 (50)	4 (7)	26 (35)	17 (45)
III / IV	118 (67)	2 (33)	53 (93)	46 (62)	17 (45)
Unknown	7 (4)	1 (17)	0 (0)	2 (3)	4 (11)
ECOG performance status					
0–1	155 (89)	5 (83)	47 (82)	67 (91)	36 (95)
≥ 2	14 (8)	0 (0)	8 (14)	6 (8)	0 (0)
Unknown	6 (3)	1 (17)	2 (4)	1 (1)	2 (5)
BM involvement					
Yes	31 (18)	0 (0)	16 (28)	14 (19)	1 (3)
No	126 (72)	5 (83)	41 (72)	51 (69)	29 (76)
Unknown	18 (10)	1 (17)	0 (0)	9 (12)	8 (21)
Extranodal sites					
0–1	129 (74)	4 (67)	43 (75)	56 (76)	26 (68)
≥ 2	43 (25)	1 (17)	14 (25)	17 (23)	11 (29)
Unknown	3 (2)	1 (17)	0 (0)	1 (1)	1 (3)
Serum LDH					
Elevated	81 (46)	3 (50)	37 (65)	30 (41)	11 (29)
Normal	84 (48)	2 (33)	19 (33)	38 (51)	25 (66)
Not evaluated	10 (6)	1 (17)	1 (2)	6 (8)	2 (5)
IPI score					
Low (0–1)	66 (38)	2 (33)	12 (21)	32 (43)	20 (53)
Low-intermediate (2)	44 (25)	1 (17)	16 (28)	19 (26)	8 (21)
High-intermediate (3)	35 (20)	1 (17)	18 (32)	13 (18)	3 (8)
High (4–5)	12 (7)	0 (0)	8 (14)	4 (5)	0 (0)
Not evaluated	18 (10)	2 (33)	3 (5)	6 (8)	7 (18)
PIT score					
Low (0)	49 (28)	2 (33)	10 (18)	21 (28)	16 (42)
Low-intermediate (1)	55 (31)	1 (17)	20 (35)	25 (34)	9 (24)
High-intermediate (2)	29 (17)	1 (17)	14 (25)	12 (16)	2 (5)
High (3–4)	14 (8)	0 (0)	10 (18)	4 (5)	0 (0)
Not evaluated	28 (16)	2 (33)	3 (5)	12 (16)	11 (29)
Primary treatment regimen					
CHOP-like	133 (76)	3 (50)	57 (100)	53 (72)	20 (53)
EPOCH-like	28 (16)	1 (17)	0 (0)	10 (14)	17 (45)
ICE	6 (3)	1 (17)	0 (0)	4 (5)	1 (3)
ESHAP	1 (1)	0 (0)	0 (0)	1 (1)	0 (0)
Gemcitabine-based	4 (2)	1 (17)	0 (0)	3 (4)	0 (0)
Others	3 (2)^a^	0 (0)	0 (0)	3 (4)^a^	0 (0)
Primary treatment regimen (grouped)					
Anthracycline based	161 (92)	4 (67)	57 (100)	63 (85)	37 (97)
Non-anthracycline based	14 (8)	2 (33)	0 (0)	11 (15)	1 (3)
Others	0 (0)	0 (0)	0 (0)	0 (0)	0 (0)
Auto-SCT					
Yes	32 (18)	1 (17)	16 (28)	15 (20)	0 (0)
No	143 (82)	5 (83)	41 (72)	59 (80)	38 (100)

^a^alemtuzumab-DHAP; High-dose methotrexate/procarbazine/vincristine

There were several differences between the three cohorts of patients. The Chinese patients were younger (median age 42.5 years), had lower IPI and PIT scores, and they had a significantly higher proportion of patients with ALK-negative ALCL compared to the Singaporean and Korean cohort. With regards to treatment, half of the Chinese patients received EPOCH as first-line therapy while none of the patients in the Singapore and Korean cohort did, and fewer Chinese patients (7%) received upfront HDC/ASCT compared to the Singaporean (28%) and Korean (19%) cohort. Follow-up times in the Singapore, Korean, and Chinese cohort were also significantly different at 6.1 (95% confidence interval (CI), 4.3–7.6), 3.0 (95% CI, 2.2–4.1), and 1.6 (95% CI 1.1–2.0) years respectively.

For the entire cohort, the median OS was not reached and the median PFS was 5.5 years (95% CI, 4.0–8.2 years). The 3-year OS and PFS rates were 89% (95% CI, 82–93%) and 61% (95% CI, 52–68%), respectively and the median follow-up time was 2.7 years (95% CI, 2.2–3.2 years). The 3-year OS for ALK- negative ALCL was 100% compared to 89% (95% CI 76–95%) in PTCL-NOS, 84% (95% CI 70–02%) in AITL, and 80% (20–97%) in MEITL.

On univariate analysis, older age, higher stage, IPI, and PIT scores were significantly associated with a poorer OS. Histological subtypes also significantly correlated with OS on univariate analysis. Age, stage, IPI, and PIT scores, gender and treatment site correlated with PFS on univariate analysis. On multivariate analysis, older age and higher stage were independently prognostic for a poorer OS and older age, male gender and higher stage were independently prognostic for a poorer PFS. Interestingly, there was no evidence that primary treatment, i.e., anthracycline versus non-anthracycline-based treatment or upfront HDC/ASCT correlated with PFS or OS on univariate and multivariate analyses. Even when we considered only patients below 60 years of age, upfront HDC/ASCT did not appear to be associated with a better OS or PFS (Table 2).

**Table 2:** Univariate and multivariate analyses of overall survival and progression-free survival

Variable		Overall survival					Progression-free survival			
	No. of events/patients	Univariate analysis		Multivariate analysis^a^		No. of events/patients	Univariate analysis	Multivariate analysis^a^		
		Hazard ratio (95% CI)	*p*-value	Hazard ratio (95% CI)	*p*-value		Hazard ratio (95% CI)	*p*-value	Hazard ratio (95% CI)	*p*-value
Age, per year^m^	24 / 175	1.06 (1.02, 1.10)	<0.001	1.06 (1.02, 1.10)	0.001	73 / 175	1.02 (1.01, 1.04)	0.002	1.02 (1.00, 1.04)	0.014
Age, years										
≤ 60	10 / 119	1	0.001			43 / 119	1	0.036		
> 60	14 / 56	4.17 (1.77, 9.85)				30 / 56	1.68 (1.04, 2.69)			
Sex^m^										
Female	6 / 65	0.46 (0.18, 1.15)	0.078	Not significant		19 / 65	0.43 (0.26, 0.73)	0.001	0.48 (0.26, 0.89)	0.015
Male	18 / 110	1				54 / 110	1		1	
Stage										
I	1 / 28	0.12 (0.02, 0.94)	0.034			5 / 28	0.24 (0.09, 0.61)	0.003		
II	2 / 22	0.47 (0.10, 2.10)				6 / 22	0.47 (0.19, 1.13)			
III	9 / 57	1.04 (0.43, 2.51)				25 / 57	0.89 (0.52, 1.51)			
IV	12 / 61	1				31 / 61	1			
Stage (grouped)^m^										
I / II	3 / 50	0.24 (0.07, 0.80)	0.007	0.19 (0.04, 0.87)	0.010	11 / 50	0.34 (0.18, 0.66)	<0.001	0.31 (0.14, 0.71)	0.002
III / IV	21 / 118	1		1		56 / 118	1		1	
ECOG performance status^m^										
0–1	18 / 155	1	0.070	Not significant		62 / 155	1	0.515	Not significant	
≥ 2	5 / 14	2.76 (1.02, 7.47)				8 / 14	1.29 (0.62, 2.70)			
BM involvement^m^										
Yes	9 / 31	2.02 (0.87, 4.71)	0.116	Not significant		18 / 31	1.52 (0.88, 2.64)	0.147	Not significant	
No	14 / 126	1				44 / 126	1			
Extranodal sites^m^										
0–1	17 / 129	1	0.428	Not significant		50 / 129	1	0.331	Not significant	
≥ 2	7 / 43	1.44 (0.60, 3.49)				20 / 43	1.30 (0.77, 2.18)			
Serum LDH^m^										
Elevated	16 / 81	1.97 (0.84, 4.61)	0.109	Not significant		40 / 81	1.56 (0.95, 2.56)	0.077	Not significant	
Normal	8 / 84	1				26 / 84	1			
Histological subtype^m^										
MEITL	2 / 6	1.88 (0.40, 8.84)	0.018	Not significant		5 / 6	1.89 (0.73, 4.88)	0.531	Not significant	
AITL	13 / 57	1.72 (0.73, 4.02)				26 / 57	0.95 (0.56, 1.61)			
PTCL-NOS	9 / 74	1				31 / 74	1			
ALK-negative ALCL	0 / 38	NE (no events)				11 / 38	0.81 (0.40, 1.62)			
IPI score										
Low (0–1)	5 / 66	1	0.023			17 / 66	1	0.024		
Low-intermediate (2)	7 / 44	2.37 (0.75, 7.51)				19 / 44	1.97 (1.02, 3.79)			
High-intermediate (3)	6 / 35	3.07 (0.93, 10.16)				18 / 35	2.31 (1.18, 4.49)			
High (4–5)	5 / 12	7.45 (2.08, 26.62)				8 / 12	2.89 (1.24, 6.72)			
PIT score										
Low (0)	2 / 49	1	0.010			10 / 49	1	0.027		
Low-intermediate (1)	9 / 55	4.53 (0.97, 21.15)				24 / 55	2.40 (1.15, 5.03)			
High-intermediate (2)	5 / 29	4.75 (0.92, 24.58)				15 / 29	2.76 (1.24, 6.17)			
High (3–4)	6 / 14	11.64 (2.31, 58.69)				8 / 14	2.88 (1.13, 7.33)			
Primary treatment regimen^m^										
Anthracycline based	22 / 161	1	0.801	Not significant		68 / 161	1	0.479	Not significant	
Non-anthracycline based	2 / 14	0.83 (0.20, 3.56)				5 / 14	0.73 (0.29, 1.81)			
Autologous SCT^m^										
Yes	3 / 32	0.41 (0.12, 1.38)	0.110	Not significant		16 / 32	0.99 (0.56, 1.74)	0.972	Not significant	
No	21 / 143	1				57 / 143	1			
Autologous SCT in young patients only (≤60 years)										
Yes	2 / 29	0.57 (0.12, 2.72)	0.457			14 / 29	1.39 (0.73, 2.65)	0.324		
No	8 / 90	1				29 / 90	1			

^a^ Final multivariable model after application of variable selection procedure. Variables used in the selection procedure are marked^m^

Note that non-proportional hazards were detected for age and stage (grouped) in univariate analysis for PFS

*AITL* angioimmunoblastic T-cell lymphoma, *ALCL* anaplastic large cell lymphoma, *ALK* anaplastic lymphoma kinase, *BM* bone marrow, *CI* confidence interval, *MEITL* monomorphic epitheliotropic intestinal T-cell lymphoma, *ECOG* Eastern Cooperative Oncology Group, *IPI* International Prognostic Index, *LDH* lactate dehydrogenase, *NA* not applicable, *NE* not evaluable, *OS* overall survival, *PFS* progression-free survival, *PIT* Prognostic Index for T-cell lymphoma, *PTCL-NOS* peripheral T-cell lymphoma, not otherwise specified, *SCT* stem-cell transplantation

We further analyzed the data excluding patients with ALK-negative ALCL since they were a low-risk population that did not undergo upfront HDC/ASCT in this study and found that an older age, male gender, advanced stage, higher PIT scores, and upfront HDC/ASCT were associated with a poorer OS on univariate analysis. However, on multivariate analysis, only age and gender were significantly associated with a poorer OS. Excluding the ALK-negative cohort, advanced age, male gender, advanced stage, elevated LDH, higher IPI, and PIT scores were associated with a poorer PFS on univariate analysis but only male gender and advanced stage were significantly associated with a poorer PFS on multivariate analysis. We also performed an analysis excluding the Chinese cohort of patients as they appeared to have a cohort of patients with more favorable parameters including a younger age, lower IPI, and PIT scores and fewer Chinese patients received upfront HDC/ASCT. In this analysis, a higher age, stage, PIT scores, and the male gender were associated with a poorer OS on univariate analysis and only a higher age and stage were significantly associated with a poorer OS on multivariate analysis. Excluding the Chinese cohort, a higher age and stage, and the male gender were associated with a poorer PFS on both univariate and multivariate analyses.

The retrospective nature of this study means patient and treatment factors, such as first-line chemotherapy regimens, are not standardized. Data on dose intensity and density are also limited. Although most of the histological diagnoses were made by hematopathologists at tertiary academic centers, there was no central review and diagnoses were made according to the World Health Organization (WHO) guidelines, which have undergone changes over the years. Small numbers of patients also limits the subgroup analysis and the manner in which end of treatment assessments were performed was variable according to institutional standards. Nonetheless, given the rarity of this disease, this study provides valuable information from large and longitudinal data sets from three major academic centers in Asia and it reflects real-world experience.

In conclusion, our study shows that patients with PTCL who do achieve CR1 may have a better prognosis than their contemporaries who do not achieve CR1. Similar to other reports^11–13^, the use of HDC/ASCT in our study did not appear to improve PFS or OS. Prospective trials are needed to validate this observation.
